# Phenotypic correlates between clock genes and phenology among populations of Diederik cuckoo, *Chrysococcyx caprius*

**DOI:** 10.1002/ece3.70117

**Published:** 2024-07-31

**Authors:** L. S. Le Clercq, V. Phetla, S. T. Osinubi, A. Kotzé, J. P. Grobler, D. L. Dalton

**Affiliations:** ^1^ South African National Biodiversity Institute Pretoria South Africa; ^2^ Department of Genetics University of the Free State Bloemfontein South Africa; ^3^ FitzPatrick Institute of African Ornithology University of Cape Town Cape Town South Africa; ^4^ School of Health and Life Sciences Teesside University Middlesbrough UK

**Keywords:** *Adcyap1*, avian, brood parasitism, *Chrysococcyx caprius*, circannual, *Clock*, cuckoo, intra‐African, migration

## Abstract

The Diederik cuckoo, *Chrysococcyx caprius*, is a small Afrotropical bird in the family Cuculidae. It is taxonomically related to 13 other species within the genus *Chrysococcyx* and is migratory in sub‐Saharan Africa. It has a unique breeding behaviour of being a brood parasite: Breeding pairs lay their eggs in the nests of a host species and hatchlings expel the eggs of the host species. The aim of the present study was to investigate diversity in two circadian clock genes, *Clock* and *Adcyap1*, to probe for a relationship between genetic polymorphisms and their role in circannual timing and habitat selection (phenology) in intra‐African migrants. DNA extracted from blood was used for the PCR amplification and sequencing of clock genes in 30 Diederik cuckoos. Three alleles were detected for *Clock* with similar genotypes between individuals from the Northern and Southern breeding ranges while 10 alleles were detected for *Adcyap1*, having shorter alleles in the North and longer alleles in the South. Population genetic analyses, including allele frequency and zygosity analysis, showed distinctly higher frequencies for the most abundant *Clock* allele, containing 10 polyglutamine repeats, as well as a high degree of homozygosity. In contrast, all individuals were heterozygous for *Adcyap1* and alleles from both regions showed distinct differences in abundance. Comparisons between both clock genes and phenology found several phenotypic correlations. This included evidence of a relationship between the shorter alleles and habitat selection as well as a relationship between longer alleles and timing. In both instances, evidence is provided that these effects may be sex‐specific. Given that these genes drive some of the synchronicity between environments and the life cycles of birds, they provide valuable insight into the fitness of species facing global challenges including climate change, urbanisation and expanding agricultural practices.

## Graphical abstract

The Diederik cuckoo is a small Afrotropical bird in the Cuculidae family that has a wide geographic distribution in sub‐Saharan Africa. A study on 30 Diederik cuckoos revealed genetic diversity in *Clock* and *Adcyap1* circadian clock genes, with distinct North–South variations in allele lengths. Phenotypic correlations indicated relationships between allele lengths, habitat selection and timing, in a potentially sex‐specific manner, offering valuable insights into the species' adaptation to global challenges.

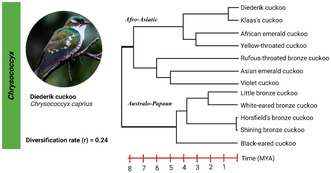

## Samevatting

Die Diederik koekoek, *Chrysococcyx caprius*, is ’n klein Afrotropiese voël in die Cuculidae familie. Dit is taksonomies verwant aan dertien ander spesies in die genus *Chrysococcyx* en is ’n migrant in sub‐Sahara Africa met die unieke teling fenologie van broeisparasietisme (broeipare lê nul eiers in die neste van gasheerspesies en kuikens verwyder die eiers van hul gashere). Die doelwit van die huidige studie is om diversiteit in twee gene van die sirkadiese klok, *Clock* en *Adcyap1*, te ondersoek om moontlike verwantskappe tussen polimorfisme en fenologie soos tydsberekening en habitat keuse te ontleed. DNS vanaf bloed monsters was gebruik vir PKR amplifikasie en reeksbepaling in dertig Diederik koekoek. Drie allele was bespeur vir die *Clock* geen, met soortgelyke genotipes onder individue van beide die Noordelike en Suidelike teling streke, terwyl tien allele bespeur is vir die *Adcyap1* geen, met korter allele in die Noord en langer allele in die Suid. Genetiese analise van die populasies, insluitend alleel frekwensie en sigositeit, het kenmerkend hoër frekwensies getoon vir die mees algemene *Clock* geen, met tien poli‐glutamien herhalings, sowel as ‘n hoë mate van homosigositeit. In teenstelling was alle individue heterosigoties vir die *Adcyap1* geen met afsonderlike verskille in mate. Vergelykings tussen beide gene en fenologie het verskeie korrelasies ontbloot. Onder andere is tekens vir ‘n verwantskap tussen korter allele en habitat seleksie sowel as langer allele en tydsberekening gevind. In beide gevalle is verdere bewyse gelewer dat die effek seks‐spesifiek mag wees. Aangesien hierdie gene die ritmiek van jaarlikse lewensiklusse reguleer verskaf hul belangrike insig tot die fiksheid van spesies wat globale uitdagings die hoof bied.

## 1 INTRODUCTION

Birds in environments that show seasonal changes in temperature (Pancerasa et al., 2018) and food availability (Stephan, 2002) have developed complex annual cycles to carefully time life events including migration and breeding (Cassone, 2014; Cassone & Westneat, 2012). Several heredity studies have also shown high correlations for intergenerational resemblance of attributes related to migration and breeding; with heredity scores (*h* ^2^) ranging between 0.38 and 0.67 in several passerine migratory species (Justen & Delmore, 2022). Despite the high heritability, related studies have also indicated that migratory species have been subject to many adaptations to their life history including changes in their departure and arrival times (Jenni & Kéry, 2003; Rubolini et al., 2007; Saino et al., 2009), staging sites (Verhoeven et al., 2018) and ranges (de Vos & Cherry, 2017) in response to environmental change. This has highlighted that while migratory avian species show marked heritability in their annual life events, some degree of adaptability may be required for a species persistence.

Due to the high heritability of these traits, it is thus likely that they are under genetic control and that genetic diversity within key genes confer an element of adaptability to the environment. Genetic studies have linked variability within genes associated with the circadian clock, such as the mean polymorphic repeat number in an exon of the *Clock* gene and 3′‐UTR of the *Adcyap1* gene, to several aspects of the annual life events of birds (reviewed in Le Clercq, Bazzi, et al., 2023e; Le Clercq, Bazzi, et al., 2023f). This included aspects such as timing of autumn migration (Justen et al., 2022; Pulido et al., 2001), timing of spring migration (Justen & Delmore, 2022; Krist et al., 2021) and breeding latitude (Johnsen et al., 2007). Thus far, these observations have been most widely illustrated in passerine (order: Passeriformes) and songbird species (Le Clercq, Bazzi, Cecere, et al., 2023) and has largely focused on species from Europe and North America.

Cuckoos present a unique case study because, in addition to being migratory species, cuckoos in the order Cuculiformes follow the unique breeding behaviour of being a brood parasite—adapting not only to their environments but also the species they parasitise. Different species of cuckoos differentially parasitise different host species (Brooker & Brooker, 1990) where cuckoo breeding pairs lay their eggs in the nests of a host species and hatchlings expel the eggs or hatchlings of the host species. For example, the Australian Horsfield's bronze cuckoo, *Chrysococcyx basalis* [Horsfield, 1821], parasitises resident populations of Superb fairy‐wren (Langmore et al., 2008), *Malurus cyaneus* [Ellis, 1782], while the Common cuckoo, *Cuculus canorus* [Linnaeus, 1758], parasitises several resident as well as short and long distance migrants (Saino et al., 2009). Due to this ‘evolutionary arms race’ most cuckoos need to be highly synchronised to their host species and follow a pattern of co‐evolution (Rönkä et al., 2022) with their hosts that results in host‐specific races; which differ in several phenotypic and behavioural attributes without constituting definitive subspecies but are rather more similar to ecotypes (Langmore et al., 2008; Reed, 1968; Skead, 1952; Soler & Soler, 2000). These races exhibit phenotypical variation that includes differences in egg characteristics such as coloration (Caves et al., 2015; Reed, 1968) and shell density (Spottiswoode, 2010), behavioural adaptations, such as the mimicking of different host sounds and behaviour (Langmore et al., 2008; Reed, 1968), persistent shared ectoparasite profiles (Lindholm et al., 1998), and high synchronicity in timing of migration and breeding (Douglas et al., 2010).

**FIGURE 1:**
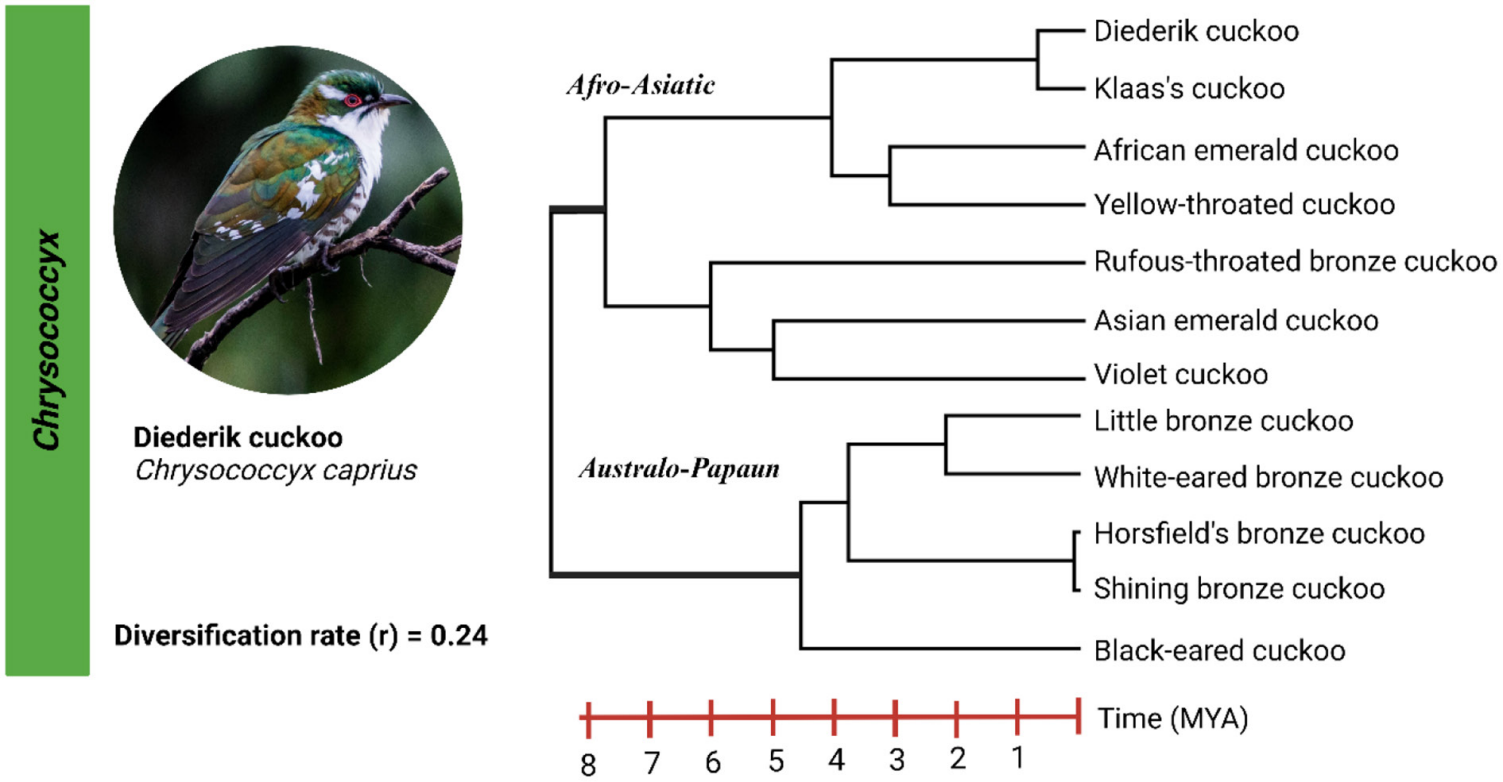
Time calibrated phylogenetic tree for species in the genus *Chrysococcyx*. The species tree was downloaded from the Bird Tree website using the Ericson phylogeny for 500 trees (https://birdtree.org/). Image created in BioRender.com. The Diederik cuckoo partitions with Klaas's cuckoo within the upper, Afro‐Asiatic, clade of cuckoos including both the African and Asian emerald cuckoos, while the bottom clade contains Australo‐Papaun cuckoos such as the Australian Horsefield's bronze cuckoo.

The Diederik cuckoo, *Chrysococcyx caprius* (Boddaert, 1783), is a small Afrotropical bird in the family *Cuculidae* (order: Cuculiformes) first described by French naturalist Georges‐Louis Leclerc, Comte de Buffon, in 1780 as part of his titular series the *Histoire Naturelle des Oiseaux* (Leclerc, 1780). It is taxonomically related to 13 other species within the genus *Chrysococcyx* (Figure 1) that diverged from other cuckoo lineages approximately 23.94 million years ago (MYA) (Jetz et al., 2012; Prum et al., 2015). The genus includes Afro‐Asiatic species such as Klaas's cuckoo, *Chrysococcyx klaas* [Stephens, 1815] and Australo‐Papuan species such as Horsfield's bronze cuckoo, *Chrysococcyx basalis* [Horsfield, 1821]. Species within the genus diverged approximately 7.71 MYA (diversification rate (*r*) = 0.24). The Diederik cuckoo is still classified as monotypic and results from detailed molecular study revealed no evidence of an emerging subspecies level divide between different populations based on neutral genomic markers and mitochondrial DNA (Smith et al., 2023). Although this species has been known for more than 300 years, little research has been conducted on them and many aspects of their life history and population structure remain uncharacterised.

**FIGURE 2:**
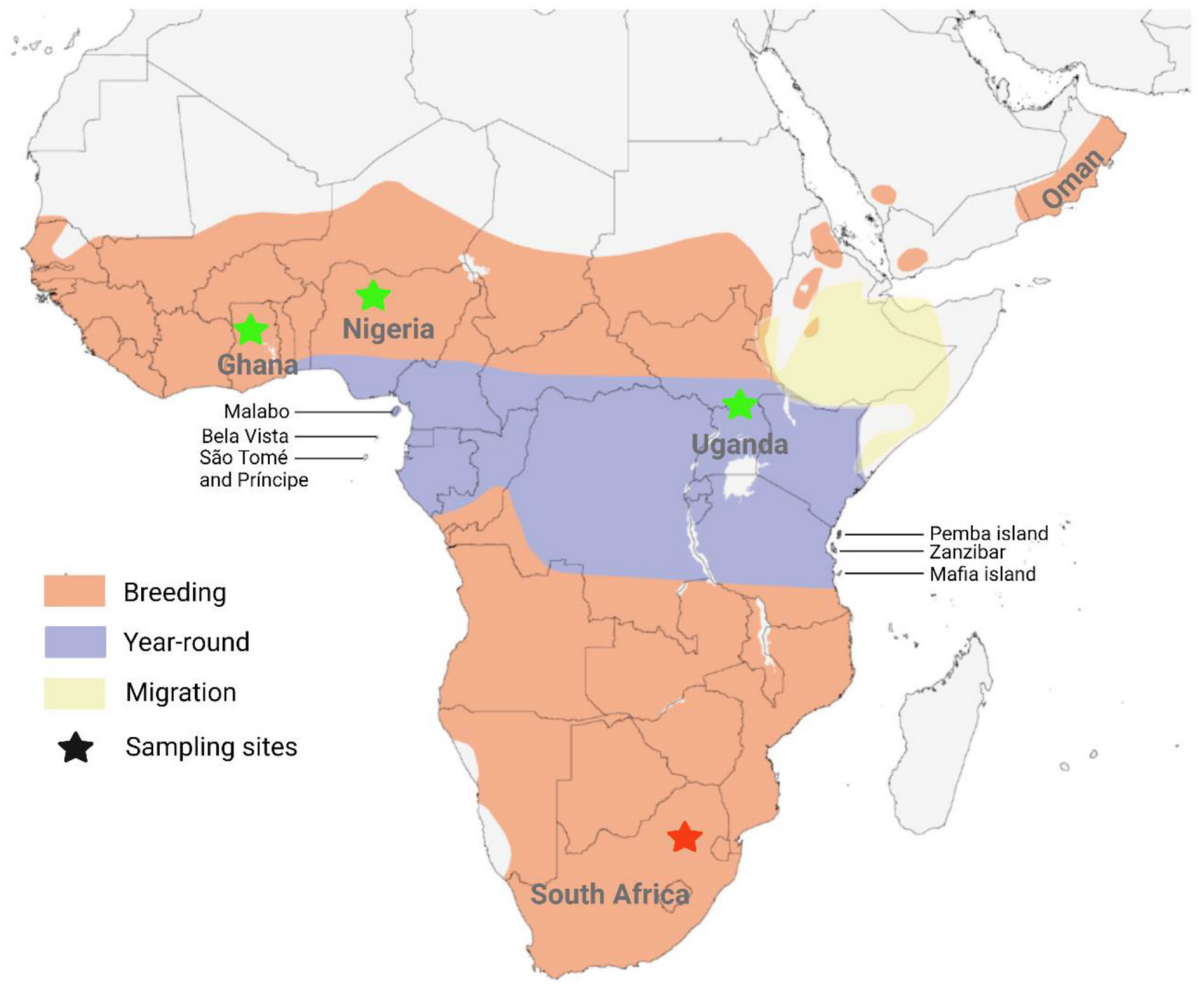
Map for the natural range of Diederik cuckoo in sub‐Saharan Africa. Regions are colour coded based on the type of habitat use. Ranges indicated in red represent habitat occupied during the local breeding season while regions indicated in purple represent areas with year‐round occupation. The small area indicated in yellow represents areas with vagrant sightings during the migration season that serves as staging sites but are typically vacated within a few days. Island populations off the West and East coast are indicated, including São Tomé and Príncipe as well as Zanzibar. The four sampling sites for this study are indicated with stars and included Ghana (green), Nigeria (green), Uganda (green) and South Africa (red). Image adapted from Birds of the World (Payne et al., 2021), created in BioRender.com.

The Diederik cuckoo has a wide distribution in sub‐Saharan Africa (Figure 2), with possible resident populations of short‐winged individuals in West Africa. The remainder are presumed to be short distance, intra‐African migrants with annual migrations between the more centrally located populations of the Equator and their Northern and Southern‐most breeding ranges, typically moving based on annual rainfall seasons (Payne et al., 2021). In their Northern ranges they typically breed between June and September. Here, they parasitise passerine birds such as the Village weaver (Gee & Heigham, 1977; Honeywell, 1971; Macdonald, 1980; Morel, 1972), *Ploceus cucullatus* [Müller, 1776], in close proximity to other weavers (Macdonald, 1980) such as Vieillot's black weaver, *P. nigerrimus* [Vieillot, 1819], and Little weaver, *P. luteolus*. [Lichtenstein, 1823]. In their Southern ranges they first arrive in September with most rapid arrivals in October before they breed as of November. Thereafter, many birds leave the breeding grounds starting in February, however, evidence from late hatching chicks indicate that some only leave in April (Hockey, 1994; Maclean et al., 1993; Rowan, 1983; Tarboton et al., 1987). In their Southern range they also parasitise the Village weaver, as well as other passerine species: Cape sparrow, *Passer melanurus* [Müller, 1776]; Cape wagtail, *Motacilla capensis* [Linnaeus, 1766]; Cape weaver, *P. capensis* [Linnaeus, 1766]; Southern masked weaver, *P. velatus* [Vieillot, 1819]; and Southern red bishop, *Euplectes orix* [Linnaeus, 1758] (de Vos & Cherry, 2017; Jensen & Jensen, 1969; Rowan, 1983). Unlike the Diederik cuckoo, most host species are year‐round resident birds that do not follow a distinct pattern of annual migration but differ in their annual timing for nesting and breeding based on hemisphere and region (Figure S1).

Because of their co‐evolution with resident species, Diederik cuckoos are particularly sensitive to changes in their host species in terms of population density (Ferguson, 1994), changing ranges (de Vos & Cherry, 2017) and changes in annual timing of life events (Saino et al., 2009), possibly leading to mismatches between the host and cuckoo species. This renders cuckoos particularly vulnerable to climate change and the decreased availability of suitable habitat due to anthropogenic activity such as expanding agricultural use and urbanisation in Africa (Greig et al., 2017; Güneralp et al., 2018). Agriculture is less likely to contribute to habitat loss in Southern Africa, as compared to Northern Africa, as widespread land reforms introduced since 1994 have resulted in a more than 10% decrease in actively cultivated land between 1994 and 2014 (Bernstein, 2013; Lidzhegu & Palamuleni, 2012; McCusker, 2004). While this has created novel opportunities for potential rewilding (Hoogendoorn et al., 2019; Navarro & Pereira, 2015), the rewilding of agricultural land after abandonment is a long and arduous process with little land recovering naturally (Carver, 2019; Lidzhegu & Palamuleni, 2012; Navarro & Pereira, 2015). Furthermore, abandoned agricultural land typically experience an initial decline in biodiversity when the habitat becomes unsuitable for insects and birds that are typically found in proximity to farms (Carver, 2019). There is, however, a significant risk in terms of urbanisation—as most counties within the range of Diederik cuckoos are experiencing significant increases in their human populations and a corresponding increase in urbanisation (Güneralp et al., 2018).

Considering migration itself is an evolutionary adaptation in response to environmental changes in temperature (Pancerasa et al., 2018), length of daylight (Robart et al., 2018) and food availability (Stephan, 2002), the ability to adapt migration strategies is crucial for the persistence of a species. Of critical importance is how the aforementioned changes might affect the habitat selection and annual rhythmicity of life events in both resident host species as well as migratory cuckoo species (Carey, 2009). At present, however, no data exist on the diversity and putative adaptive potential, driven by the possibility for selection to shift between genotypes that confer reproductive success, of Diederik cuckoo or their host species within genes that are associated with annual life events including migration. Thus, the aim of the present study is to investigate the existing diversity in two candidate clock genes, *Clock* and *Adcyap1* and probe for a relationship between genetic variance and their potential role in circannual timing in the Diederik cuckoo. This will be conducted to test several hypothesis including if (i) gene diversity follows a geospatial pattern by latitude and/or longitude, (ii) gene diversity relates to differences in annual timing of breeding, (iii) differences are sex‐specific and (iv) if similarity exists between genotypes of cuckoos and their most common hosts.

## 2 METHODS

### 2.1 Specimens and ethics

Protocol approval for the present study was obtained from the protocol committee of the Department of Genetics, University of the Free State (approval number: Res18/2020). Ethics approvals were obtained from the University of the Free State (approval number: UFS‐AED2020/0015/1709) as well as the South African National Biodiversity Institute (approval number: SANBI/RES/P2020/30). The appropriate research permit was also obtained from South African regulatory authorities, specifically the Department of Agriculture, Land Reform, and Rural Development (Section 20 permit: 12/11/1/1/18 (1824JD)). Specimens suited to the present study (*N* = 30) were identified from existing catalogues of samples collected for the Diederik cuckoo (Figure 1), including *C. caprius* lineages from West Africa (Ghana and Nigeria) and East Africa (Uganda) and *C. caprius* from South Africa (Table 1 and Figure 2). Samples were partitioned based on subspecies, sex, migration timing and location. Sex was determined by molecular methods as previously described (Smith et al., 2023) and 21 males and nine females were assayed. Furthermore, samples were partitioned into birds who experienced increased activity early or late during their annual breeding cycles. This was based on time of capture and sampling information from field data, as birds actively breeding or laying eggs tend to remain close to their nesting site while those who have completed these tasks move away from nesting sites and could easily be caught. Furthermore, it is known that some individuals schedule their migration earlier while others migrate later, this likely is reflected in their timing of breeding. Sample details including National Centre for Biotechnology Information (NCBI) BioSample numbers (SAMN31832894–922) are listed in Table S1.

**TABLE 1:** Locations, including latitude and longitude, sample sizes (*N*) and breeding seasons for specimens used in the present study.

Location	Latitude	Longitude	*N*	Season
East Africa:				
Uganda	N 9°43'4.79"	E 7°31'55.20"	3	Jun–Sept (North)
West Africa:				
Ghana	N 9°5'16.79"	W 1°48'32.4"	2	Jun–Sept (North)
Nigeria	N 9°52'40.8"	W 8°58'26.4"	8	Jun–Sept (North)
Southern Africa:				
South Africa	S 23°3'21.6"	E 29°8'59.99"	17	Nov–Apr (South)

### 2.2 DNA extraction, polymerase chain reaction (PCR) and sequencing

DNA was extracted from 100 μL of whole blood with the commercially available Quick‐DNA™ Miniprep Plus Kit (Zymo Research, Irvine, California, United States), according to manufacturer's instructions. The final step eluted purified DNA to approximately 25 μL of pure DNA for subsequent analysis. The DNA concentration and purity was determined by spectrophotometric measurement of 1 μL extract applied to the Nanodrop™ One (Thermo Fisher Scientific, Waltham, Massachusetts, USA) instrument. This was determined by measuring the absorbance at wavelengths of *A*_260_ and *A*_280_ to calculate the *A*_260_/*A*_280_ ratio to determine purity in addition to concentration in ng/μL.

DNA was amplified by short‐range PCR for both the *Clock* (NCBI Gene ID: 9575) and *Adcyap1* (NCBI Gene ID: 116) genes as previously described (Le Clercq et al., 2023d). Briefly, the PCR reaction were set up with 2 μL of DNA and 1.5 μL of each forward or reverse primer respectively (final concentration of 0.2 μM), were added to 15 μL of EmeraldAmp® GT PCR 2× Master Mix (Takara Bio Inc., Kusatsu, Shiga Prefecture, Japan) and adjusted to a final reaction volume of 25 μL with sterile water. Reactions were subjected to thermal cycling on the SimpliAmp™ Thermal Cycler (Thermo Fisher Scientific, Waltham, Massachusetts, USA) as follows: Initial denaturation at 98°C for 10 s, denaturation and annealing at 52°C (*Adcyap1*) or 55°C (*Clock*) for 30 s and elongation for 1 min at 72°C for 40 cycles; followed by a final hold at 4°C. Following PCR, amplicons were resolved by electrophoresis on a 4% TAE‐agarose gel, for 1 h 30 at 60 V, along with the Quick‐Load® Purple 100 bp DNA ladder (New England Biolabs, Ipswich, Massachusetts, United States) to confirm successful amplification and resolve possible alleles (Bhattacharya & Van Meir, 2019). PCR products were purified using Exonuclease I (Exo)/Shrimp Alkaline Phosphatase (SAP; Thermo Fisher Scientific, Waltham, Massachusetts, USA) following manufacturer's instructions. Standard reaction chain termination sequencing procedures were followed (Le Clercq et al., 2023c) individually for both the *Clock* and *Adcyap1* PCR products using the BigDye™ Terminator v. 3.1 Cycle Sequencing Kit (ABI, Thermo Fisher Scientific, Waltham, Massachusetts, USA). As per manufacturer's recommendations, 10–30 ng of PCR product was used in a 10 μL reaction with the same primers as before (final concentration of 3.2 pmol). Sequence reactions were cleaned with the BigDye XTerminator™ Purification Kit (ABI, Thermo Fisher Scientific, Waltham, Massachusetts, USA) protocol using 45 μL of SAM™ solution and 5 μL BigDye XTerminator™ bead solution. Sequence reactions were analysed by capillary electrophoresis on the 3500 Genetic Analyser (ABI, Thermo Fisher Scientific, Waltham, Massachusetts, USA) and the Seq Scanner 2 software (ABI, Thermo Fisher Scientific, Waltham, Massachusetts, USA).

### 2.3 Supplementary data

Data were supplemented by retrieving additional sequence data from the NCBI sequence read archive (SRA) for host species of the Diederik cuckoo, including specimens for Village weaver (SRR17013387) and Cape wagtail (Table S2). Data from whole genome sequencing experiments were retrieved with the SRA toolkit version 3.0.0 (https://github.com/ncbi/sra‐tools) in FASTQ format and aligned in Geneious Prime 2022 (www.geneious.com) to reference sequences for the *Clock* and *Adcyap1* genes, respectively. The aligned reads for each gene were exported in FASTA format and the diploid alleles for the microsatellite repeats determined using the Perl script MEGASAT (Zhan et al., 2017) version 1.0, with Perl implemented in Strawberry Perl (64‐bit) version 5.32.1.1 (https://strawberryperl.com/), or through visual inspection. Moult data were compiled from the available literature (Hanmer, 1980; Ramudzuli, 2021). Phylogenetic data for *Chrysococcyx* cuckoos were retrieved from the Bird tree website (www.birdtree.org) using the Ericson phylogeny (Jetz et al., 2012). Phylogenetic trees were summarised using TreeAnnotator 2.6.3, part of BEAST 2.6.3 (Bouckaert et al., 2014), to a 60% consensus tree with a 10% burn in. Time trees and divergence times were retrieved from the Time Tree resource (Kumar et al., 2022) using PAReTT version 1.0.2 (Le Clercq, 2023a; Le Clercq, Kotzé, Grobler, & Dalton, 2023) and were used to calibrate the phylogenetic tree as well as compute diversification rates.

### 2.4 Data analysis

Sequence data output was aligned using the MAFFT version 7 multiple and pair‐wise aligner (Katoh & Standley, 2013) followed by analysis in BioEdit version 7.2.6.1 (Hall, 2011). The *Clock* gene alleles were transformed to represent only the poly‐Q repeat as previously described (Le Clercq, Bazzi, Cecere, et al., 2023). Population genetics analyses were done using POPGENE 1.32 (Yeh et al., 1997) to test for Hardy–Weinberg equilibrium (Hardy, 1908; Weinberg, 1908), based on the equilibrium equation (Equation 1). Here, the equilibrium is expressed as the sum of the squared homozygous genotypes and the product for homozygous genotypes multiplied by two. In the formula, p represents the first allele, q represents the second allele, and r represents the third allele.

(1) $$p^{2}+q^{2}+r^{2}+2\mathrm{pq}+2\mathrm{pr}+2\mathrm{qr}=1$$

Both the chi‐squared (*χ* ^*2*^) and maximum likelihood tests were used to assess deviation from equilibrium (with significance measured at *α* = 0.02). The same programme was also used to calculate allele frequencies as well as the observed (*H*_o_) and expected (*H*_e_) heterozygosity, based on the standard equation (Equation 2) with slight modification (Levene, 1949). Here, the expected heterozygosity is expressed as the difference between one and the sum of squared allele frequencies (*p*) from the *i*th allele to the *k*th allele. Based on the three‐allele system, this corresponds to *p*, *q* and *r* alleles in the equilibrium equation.

(2) $$\text{Expected heterozygosity}\;\left(H_{e}\right)=1–\sum_{i=1}^{n=k}{p_{i}}^{2}$$

Lastly, the number of migrants (*N*_m_) between the two populations was calculated (Equation 3) using the fixation index (*F*_ST_). Here, the difference between one and the *F*_ST_ is multiplied by a quarter and subsequently divided by the *F*_ST_.

(3) Number of migrantsNm=0.251−FSTFST

Population structure was accessed using both alleles in STRUCTURE 2.3.4 using the admixture model with populations used as priors (Pritchard et al., 2000). The parameter set had a burn‐in of 500 and was run with 10,000 repeats to achieve coalescence. Experiments were run testing a population number (*K*) between 1 and 10 with a hundred iterations per K. The final results were exported as a zipped archive and the best structural model extracted using the web interface of STRUCTURE HARVESTER (Earl & vonHoldt, 2012). The first allele, second allele and average were compared to timing (sample date) and location (latitude and longitude) in R version 4.0.2 (R Core Team, 2020) by performing individual linear models using a Least Squares model. Models were fitted using the full data sets as well as with subset analyses based on sex to test for potential sex‐linked effects.

## 3 RESULTS

### 3.1 Gene alleles and population genetics

**FIGURE 3:**
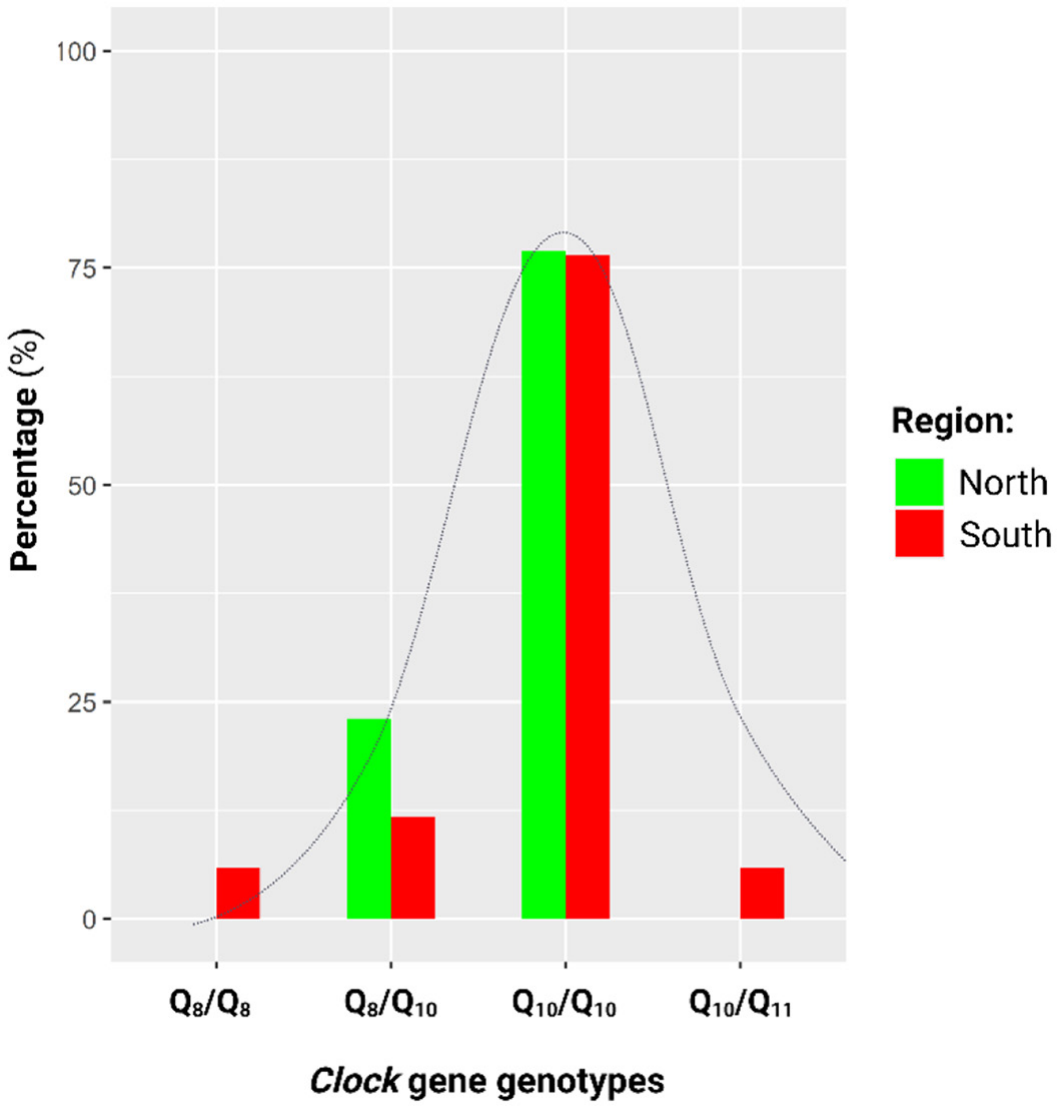
Genotype distributions for the *Clock* gene in the Northern (green) and Southern (red) populations. In both populations the most common genotype was individuals homozygous for the Q_10_ allele. Furthermore, heterozygous individuals for Q_8_ and Q_10_ were detected in both populations. The Northern population had two genotypes (Gt) and no private alleles. The Southern population had slightly higher genetic diversity evidenced by four genotypes, including a heterozygous individual for Q_10_ and the private allele Q_11_. The grey bell‐shaped normal curve was fitted over the distributions and shows evidence of purifying or stabilising selection. Graphs were created in R and the image created in BioRender.com.

Three alleles were detected in total for the *Clock* gene, corresponding to 8 (Q_8_), 10 (Q_10_), and 11 (Q_11_) poly glutamine repeats (Table 2). For the Northern population, only two alleles were present with frequencies of 0.115 (Q_8_) and 0.885 (Q_10_) respectively. Tests for deviation from equilibrium, as measured by Hardy–Weinberg, were not significant (*χ* ^*2*^ = 0.142, df = 1, *p* = .71). In the Northern population, the observed homozygosity was 0.769, while the calculated expected homozygosity was 0.788 for the *Clock* gene; the observed heterozygosity (*H*_o_) was 0.231 while the calculated expected heterozygosity (*H*_e_) was 0.212 for the *Clock* gene. For the Southern population, the same two alleles (Q_8_ and Q_10_) were detected, at frequencies of 0.118 (Q_8_) and 0.853 (Q_10_), respectively, while a third allele (Q_11_) was detected in one individual (frequency of 0.029). As for the Northern population, the tests for deviation from equilibrium in the Southern population were not significant (*χ* ^*2*^ = 4.513, df = 3, *p* = .21). In the Southern population, the observed and expected homozygosity were 0.824 and 0.734 respectively, while the observed (*H*_o_) and expected heterozygosity (*H*_e_) was 0.177 and 0.266. In both populations, most of the homozygous individuals were of the Q_10_/Q_10_ genotype (Figure 3), however, one individual from South Africa was homozygous for the Q_8_ allele (Q_8_/Q_8_). Heterozygous individuals included those with the Q_8_/Q_10_ genotype, detected in both Northern and Southern populations, as well as a rare Q_10_/Q_11_ genotype detected in one individual from South Africa.

**TABLE 2:** Allele frequencies and population genetics results for *Clock* and *Adcyap1* genes, including Hardy–Weinberg (HW) and heterozygosity (*H*), for Diederik cuckoo populations.

Pop	*N*	*Clock* gene						*Adcyap1* gene												
		Q_8_	Q_10_	Q_11_	*H*_o_	*H*_e_	HW	142	144	146	148	150	152	154	156	158	160	*H*_o_	*H*_e_	HW
North	26	0.115	0.885	–	0.231	0.212	0.71	0.039	0.115	0.115	0.039	0.115	0.154	0.115	0.115	0.192	–	1.00	0.91	0.58
South	34	0.118	0.853	0.029	0.177	0.266	0.21	0.031	–	–	0.094	0.188	0.125	0.094	0.188	0.094	0.188	1.00	0.88	0.07

*Note*: For the *Clock* gene, two alleles (Q_8_ and Q_10_) were detected for specimens from the Northern range while three alleles (an additional Q_11_ allele) were detected for specimens from the Southern range. In the Northern population, the observed heterozygosity (*H*_o_) was higher than the expected heterozygosity (*H*_e_) while the inverse was true for the Southern population. In both populations, the *p*‐value for Hardy–Weinberg was not significant and as such there was no evidence for deviation from equilibrium. For the *Adcyap1* gene, nine alleles were detected in the Northern population with 152 and 158 bp being most abundant, while seven alleles were detected for the Southern population with 150, 156 and 160 bp being the most abundant. Both populations were completely heterozygous with the Northern population passing equilibrium tests while the Southern population, still in equilibrium (*p* < .05), was close to deviation.

**FIGURE 4:**
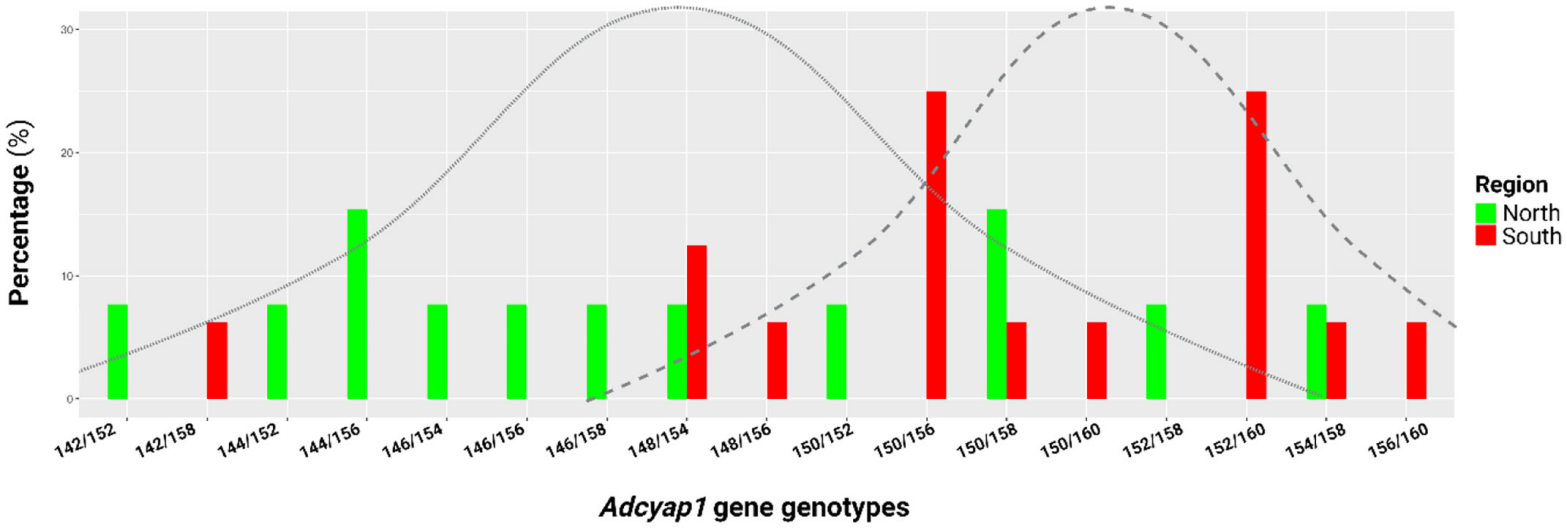
Genotype distributions for the *Adcyap1* gene in the Northern (green) and Southern (red) populations. In the Northern populations the most common genotypes were heterozygous 144/156 and 150/158, however, 11 genotypes (Gt) were detected in total with two private alleles (PA). The Southern population had slightly lower genetic diversity evidenced by nine genotypes, with only one private allele. The two most common genotypes were 150/156 and 152/160. The grey bell‐shaped normal curve was fitted over the distributions and shows evidence of disruptive selection. Graphs were created in R and the image created in BioRender.com.

Ten alleles were detected for the *Adcyap1* gene (Table 2) ranging from 142 base pairs (bp, 22 bp repeat) to 160 bp (40 bp repeat). In the Northern population, nine alleles were detected of which two represented private alleles of 144 and 146 base pairs, respectively. Five alleles were present at a frequency of 0.115 while the two most common alleles were 152 and 158 bp with a frequency of 0.154 and 0.192 respectively. Tests for deviation from equilibrium, as measured by Hardy–Weinberg, were not significant (*χ* ^*2*^ = 33.67, df = 36, *p* = .58). All individuals were heterozygous while the calculated expected heterozygosity was 0.91. The Southern population had eight alleles of which one allele (160 bp) was private to this region. Three alleles of 150, 156 and 160 bp represented the most abundant alleles, present at a frequency of 0.188 each. Tests for deviation from equilibrium by Hardy–Weinberg, detected a significant departure from equilibrium (*χ* ^*2*^ = 51.17, df = 28, *p* = .01). As for the Northern population, all individuals were heterozygous with an expected heterozygosity of 0.88. Due to the high number of alleles, a total of 17 genotypes were detected (Figure 4). In the Northern population, nine genotypes were present at a near equal number apart from two genotypes, 144/156 and 150/158 bp, which had additional individuals. The Southern population had nine genotypes of which the two most common genotypes were 150/156 and 152/160 bp respectively with each having four individuals in total. Eight of the genotypes detected in the Northern population were not detected in the Southern population while six genotypes detected in the Southern population were absent from the Northern population; only three genotypes were conserved in both populations.

**FIGURE 5:**
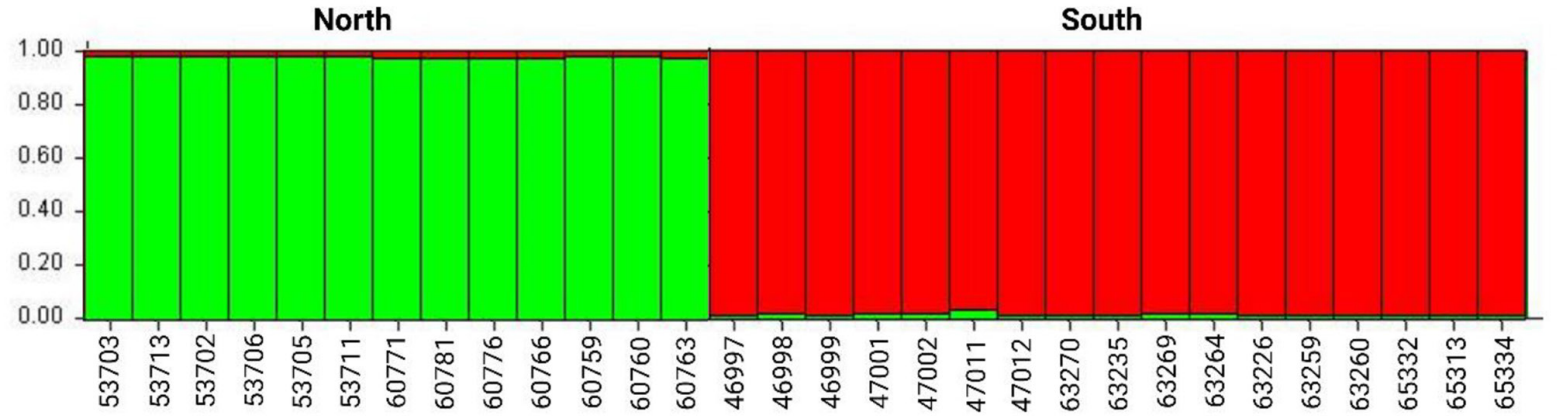
Bar plot of results from STRUCTURE analyses based on genotypes of two candidate genes, *Clock* and *Adcyap1*, showing the fraction of population identity on the y‐axis and individual samples, grouped by region, on the x‐axis. A two‐population model was detected as the optimal descriptor of population structure with clear partitioning between the Northern (green) and Southern (red) populations, however, low levels (<1%) of admixture were still detected.

Based on the *Clock* gene approximately 123 migrants (*N*_m_) were estimated per generation, while calculations based on the *Adcyap1* gene estimated at least 10 migrants (*N*_m_) per generation. Population structure analyses, performed with STRUCTURE, supported a two‐population model (*K* = 2) as the best explanatory model for the observed genetic diversity using both the *Clock* and *Adcyap1* genes (Figure 5). The results indicated a high degree of similarity between individuals from the North, indicated in green, and the South, indicated in red. A low level of admixture was observed between for both populations.

### 3.2 Relationship between genes and location

Linear models fitted between the first or second alleles for the *Clock* gene, as well as the average or mean repeat length, and measures of locality such as latitude, longitude, and region did not yield any significant results for the total sample set (Table 3). A similar absence of correlation existed when fitting models using the two alleles and their mean length for males. There was, however, a significant correlation with both latitude (*F* = 4.41, df = 6, *R* ^2^ = .42, *p* = .08) and longitude (*F* = 4.49, df = 6, *R* ^2^ = .43, *p* = .08) for females in models fitted for the first, shorter allele. Similar correlates were, however, absent for the second, longer allele. This same trend was still present when comparing mean allele size to both latitude (*F* = 3.38, df = 6, *R* ^2^ = .40, *p* = .09) and longitude (*F* = 4.37, df = 6, *R* ^2^ = .42, *p* = .08). When comparing alleles to region, as either North or South, a correlation was also detected in relation to the length of the first allele (*F* = 4.50, df = 6, *R* ^2^ = .43, *p* = .08) as well as the mean allele length (*F* = 4.32, df = 6, *R* ^2^ = .42, *p* = .08).

**TABLE 3:** Results for individual linear models between the *Clock* or *Adcyap1* alleles and phenology, fitted in *R*.

Linear model	*Clock* gene	*Adcyap1* gene
Allele 1:		
Latitude	*F* = 4.41, *R* ^2^ = .42* (♀)	*F* = 2.97, *R* ^2^ = .10*
		F = 4.69, *R* ^2^ = .44* (♀)
Longitude	*F* = 4.49, *R* ^2^ = .43* (♀)	*F* = 4.78, *R* ^2^ = .15**
		*F* = 4.91, *R* ^2^ = .45** (♀)
Date	–	F = 4.13, *R* ^2^ = 0.18** (♂)
Region	*F* = 4.50, *R* ^2^ = .43* (♀)	*F* = 5.33, *R* ^2^ = .17**
		*F* = 5.06, *R* ^2^ = .46** (♀)
Allele 2:		
Latitude	–	*F* = 2.84, *R* ^2^ = .10*
Longitude	–	*F* = 11.09, *R* ^2^ = .29**
		*F* = 8.65, *R* ^2^ = .31** (♂)
Date	*F* = 5.08, *R* ^2^ = .15**	–
	*F* = 3.95, *R* ^2^ = .17* (♂)	
Region	–	*F* = 5.74, *R* ^2^ = .18**
		*F* = 3.52, *R* ^2^ = .16* (♂)
Mean allele:		
Latitude	*F* = 3.38, *R* ^2^ = .40* (♀)	*F* = 3.75, *R* ^2^ = .12*
Longitude	*F* = 4.37, *R* ^2^ = .42* (♀)	*F* = 9.17, *R* ^2^ = .25**
		*F* = 5.28, *R* ^2^ = .23** (♂)
Date	–	*F* = 4.33, R^2^ = .19** (♂)
Region	*F* = 4.32, *R* ^2^ = .42* (♀)	*F* = 7.23, *R* ^2^ = .21**
		*F* = 3.57, *R* ^2^ = .38* (♀)

*Note*: Models were fitted for the first, shorter allele (allele 1) and second, longer allele (allele 2) as well as the average allele size. Only those models that showed a significant correlation are indicated. Both the *F*‐test value (*F*) and the correlation coefficients (*R* ^2^) for the models are indicated as well as the group for which significance was achieved as either all, male (♂) or female (♀). Significance: *p ≤ .10, **p ≤ .05.

Comparison between allele size of *Adcyap1* and latitude, longitude, as well as regions detected a significant relationship for both the shorter, longer and mean allele (Table 3). For the first, shorter, allele a relationship with latitude was detected for both the full data set (*F* = 2.97, df = 27, *R* ^2^ = .10, *p* = .09) as well as the separate analysis of females only (*F* = 4.69, df = 6, *R* ^2^ = .44, *p* = .07) while a similar relationship existed for the second, longer, allele for the full data set (*F* = 2.84, df = 27, *R* ^2^ = .10, *p* = .10). This relationship was still evident when analysing the mean allele size for the full data set (*F* = 3.75, df = 27, *R* ^2^ = .12, *p* = .06), although sex‐specific effects were not detected. Correlations also existed between longitude and allele size for the first (*F* = 4.78, df = 27, *R* ^2^ = .15, *p* = .04), second (*F* = 11.09, df = 27, *R* ^2^ = .29, *p* = .003) and mean (*F* = 9.17, df = 27, *R* ^2^ = .25, *p* = .005) alleles. Sex‐specific analyses showed a strong correlation for the first allele (*F* = 4.91, df = 6, *R* ^2^ = .45, *p* = .07) in females as well as the second (*F* = 8.65, df = 19, *R* ^2^ = .31, *p* = .01) and mean (*F* = 5.28, df = 19, *R* ^2^ = .23, *p* = .03) allele in males. When analysing regional differences, a correlation was detected for the first allele in the full dataset (*F* = 5.33, df = 27, *R* ^2^ = .17, *p* = .03) as well as females only (*F* = 5.06, df = 6, *R* ^2^ = .46, *p* = .07). A comparable relationship was detected for the second allele using all samples (*F* = 5.74, df = 27, *R* ^2^ = .18, *p* = .02) as well as for the males only (*F* = 3.52, df = 19, *R* ^2^ = .16, *p* = .08) analyses. This trend was conserved when assessing the mean allele for the whole population (*F* = 7.23, df = 27, *R* ^2^ = .21, *p* = .01) as well as in females (*F* = 3.57, df = 6, *R* ^2^ = .38, *p* = .10).

### 3.3 Relationship between genes and timing

Models were fitted between both *Clock* alleles and their mean in relation to timing expressed as the date of capture and sampling as well as the date normalised between regions in relation to the Spring Equinox and Summer Solstice in either hemisphere. When comparing alleles for all study samples, no correlation was detected for the first allele, however, a significant correlation was observed for the second, longer allele (*F* = 5.08, df = 28, *R* ^2^ = .15, *p* = .03). This correlation was not conserved when analysing the mean allele length nor when normalising dates to either the equinox or solstice. In testing, the same models for either sex, a significant relationship was seen for the second allele in males (*F* = 5.08, df = 19, *R* ^2^ = .15, *p* = .03) but not in females (*F* = 0.50, df = 6, *R* ^2^ = .08, *p* = .50). Similar to the total population analyses, the relationship with timing was not detected when analysing the mean allele length in either males or females. Models fitted to the *Adcyap1* gene detected no relationship when analysing the total population; however, a relationship was detected when analysing only males for both the first, shorter, allele (*F* = 4.13, df = 19, *R* ^2^ = .18, *p* = .06), as well as the mean allele (*F* = 4.33, df = 19, *R* ^2^ = .19, *p* = .05).

### 3.4 Data for host species

Reference genomes are not currently available for any of the host species which Diederik cuckoos parasitise. SRA sequence data were, however, available (Table S2) for three host species: The Cape wagtail (*N* = 5), Red bishop (*N* = 5) and Village weaver (*N* = 1). BLAST searches of available sequence data from the NCBI SRA did not yield any data for the region of either gene analysed in this study for Red bishops or Cape wagtails. Therefore, data were retrieved for only one Village weaver sampled in Gabon. Based on the data retrieved from SRA, the Village weavers analysed (*N* = 1) were homozygous for the Q_8_ allele for *Clock* and heterozygous 139/141 bp (22/24 bp repeat) for *Adcyap1*.

## 4 DISCUSSION

Polymorphisms in candidate genes from the circadian clock circuitry have previously been associated with various attributes of migration phenology (Le Clercq, Bazzi, et al., 2023e; Le Clercq, Bazzi, et al., 2023f). Although a large body of evidence already exists (Le Clercq, Bazzi, Cecere, et al., 2023), thus far most studies were focused on passerine species of which only a fraction use habitat on the African continent. In this study, we used a candidate gene approach to assay polymorphisms in clock genes with putative associations to phenology based on the circadian clock, in the intra‐African migratory species Diederik cuckoo. Polymorphisms were screened for 30 samples representing three sampling sites in West, East and South Africa. From the individuals screened, three alleles were identified for the *Clock* gene, while 10 alleles were detected for the *Adcyap1* gene. Least squares linear models identified putative associations between the shorter *Clock* alleles and timing and longer alleles were associated with habitat selection as measured by latitude. Similar correlations existed for *Adcyap1* where both alleles were correlated to latitude and longitude while the shorter and mean allele was correlated to timing. Interestingly, while some correlations could be detected at the population level, many of these attributes appear to have sex‐specific contributions. This study presents the first evidence for an association between clock gene polymorphisms and phenology in a non‐passerine intra‐African migrant and provides some insight into extant genetic diversity that natural selection may act upon in response to the changing landscape of Africa.

Population genetic analyses showed distinctly higher frequencies for the most abundant *Clock* allele, Q_10_, compared with the less abundant Q_8_ allele. Due to many individuals carrying the Q_10_/Q_10_ genotype, a high level of homozygosity and low heterozygosity was detected. In the case of the Northern individuals, the observed values were lower than expected for homozygosity while higher than expected heterozygosity was detected. The inverse trend was observed for the Southern individuals. Nevertheless, the homozygosity and heterozygosity between Northern and Southern populations were close in approximation to each other. The low number of alleles and high homozygosity is indicative of purifying selection (Cvijović et al., 2018; Hughes et al., 2003) which may either relate to the fact that these polymorphisms are contained in exonic sequence or be indicative that the Q_10_ allele provides an advantage in these species (Cassidy, 2021). For the *Adcyap1* gene, more alleles and genotypes were detected for the Northern population in comparison to the Southern population; although both populations showed substantially more diversity in *Adcyap1* as compared to *Clock*. These alleles tended to be shorter in the Northern population while longer alleles were prevalent in the Southern population. This may be indicative of disruptive selection (Gross, 1985; Hendry et al., 2009) due to shorter alleles providing an advantage in Northern individuals while longer alleles are favoured in Southern individuals. Due to the large number of alleles, all individuals in both populations were heterozygous as the probability of heterozygosity increases with alleles numbers while heterozygosity itself can aid in the expansion of allele numbers (Amos et al., 2008). Concurrently, the high heterozygosity may indicate a case of heterozygote advantage (Cassidy, 2021). The expansion of allelic variants for *Adcyap1* may also be related to the polymorphism occurring in the 3′‐UTR which, as a non‐coding gene segment, is less constrained (Chen et al., 2012; Mazumder et al., 2003; Steri et al., 2018).

With the exception of the *Adcyap1* gene in the Southern population, tests of equilibrium showed no significant deviations which is indicative that allele frequencies may stay the same between generations (Hedrick, 1987) in the Northern ranges with some evidence of differentiation happening in the South; consistent with previous phylogenetic studies in this species using nuclear markers and mitochondrial DNA (Smith et al., 2023). A recent review on clock genes in migrating birds (Le Clercq, Bazzi, et al., 2023e; Le Clercq, Bazzi, et al., 2023f) found similar patterns of low heterozygosity and high homozygosity for populations in equilibrium for the *Clock* gene and higher heterozygosity for *Adcyap1*. This may indicate that the patterns of inheritance and selection in the *Clock* and *Adcyap1* genes are conserved among most avian lineages. It should be noted that heterozygosity has the potential to increase diversity at polymorphic sites (Amos et al., 2008) and, as such, the presence of heterozygous individuals in Diederik cuckoos may confer continued or improved fitness over time by providing more genotypes for natural selection to act upon in adaptive evolution (Cassidy, 2021). This will be critical given the complex landscape of expanding human populations on the African continent with likely increases in agricultural use that could encroach into the habitats of cuckoos and their hosts (Güneralp et al., 2018), possibly requiring shifts in staging sites as well as habitat selection.

Three alleles were detected for the Diederik cuckoo *Clock* gene, which is similar to species such as the Barn swallow, *Hirundo rustica* (Bazzi et al., 2015) and Eurasian hoopoe, *Upupa epops* (Bazzi, Cecere, et al., 2016), but substantially less diverse than alleles detected in warbler species (Le Clercq, Bazzi, et al., 2023e; Le Clercq, Bazzi, et al., 2023f). On the other hand, the *Adcyap1* gene had 10 alleles which is comparable to observations in several warbler species, including the Eurasian reed warbler, *Acrocephalus scirpaceus* (Bazzi, Cecere, et al., 2016), and Willow warbler, *Phylloscopus trochilis* (Bazzi et al., 2017). Although non‐passerine species remain largely data deficient and lack data for comparison among cuckoos, a previous study did explore clock gene polymorphisms in the European nightjar, *Caprimulgus europaeus* (Bazzi, Cecere, et al., 2016). Cuckoos, as part of the superorder *Otidimorphae*, and Nightjars, as part of the superorder *Strisores*, both form part of the *Otidae* clade of avian species (Jarvis et al., 2014; Prum et al., 2015). In comparison, the Diederik cuckoo has three alleles ranging from 8 to 11 poly‐Q repeats for the *Clock* gene while the European nightjar has two alleles ranging from eight to nine poly‐Q repeats. For the *Adcyap1* gene, Diederik cuckoos had 10 alleles ranging in size from 142 to 160 bp while European nightjars had nine alleles ranging from 146 to 160 bp. It is therefore likely that a range of between 8 and 10 repeats for *Clock*, and 144–160 bp for *Adcyap1*, is common within this lineage. In comparison to the passerine hosts that are parasitised by Diederik cuckoos, the single Village weaver was found to have alleles of approximately eight repeats in *Clock* and *Adcyap1* genes of approximately 139–141 bp (or 22–24 bp repeats). This overlap may indicate either possible co‐evolution or specialised selection between cuckoos and their hosts based on clock genes, consistent with previous studies on brood parasites (Soler et al., 2015; Soler & Soler, 2000).

Comparisons between both *Clock* and *Adcyap1* alleles and phenology found several phenotypic correlations. The first was evidence of a relationship between the first, shorter *Clock* alleles and both latitude as well as longitude. The second was a relationship between the first *Clock* allele and region. Interestingly, both observations were made when analysing only female individuals. For *Adcyap1*, phenotypic correlates were found in relation to both latitude and longitude as well as region. In the first, shorter, allele this relationship appeared to be largely present in females, while the correlation found for the second, longer, allele showed stronger evidence for males. This is consistent with previous findings that clock genes show strong spatial patterns in females that are generally more selective of breeding site (Liedvogel et al., 2009). Lastly, evidence of a relationship was detected for the second, longer *Clock* allele and the first, shorter, *Adcyap1* allele with timing, which was observed in males whereas the observed relationship was diminished for females. This is consistent with previous studies that have also found evidence for a sex‐specific effect of clock genes on timing (Bazzi et al., 2017). While several of these correlates had moderate statistical support, this does not abrogate their significance and could likely be improved through increased sample sizes to reduce variance.

Our analyses also addressed a pertinent question previously raised regarding the treatment of data from diploid alleles in analyses such as migration genetics (Le Clercq, Bazzi, et al., 2023e; Le Clercq, Bazzi, et al., 2023f). Studies have applied varied methods when analysing diploid polymorphic repeat data including analysing the sum of alleles, the mean of alleles or only the longer alleles. No evidence of parental imprinting, which typically results in the transcription of only one allele (Andergassen et al., 2021), was found in analyses of methylation in the clock genes of birds (Saino et al., 2019). It therefor stands that both genes are transcribed and differentially contribute to phenotypes. There is, however, no knowledge as to whether both genes are co‐dominant or if a specific allele contributes more to any given trait. For this reason, each allele was tested individually (in addition to testing mean allele length) and evidence is provided that individual alleles may affect phenotypes more so than could be detected by only analysing either the sum, mean or presumed to be dominant alleles.

Several limitations existed for the present study. Firstly, a high degree of collinearity between measures, such as latitude, longitude and date, precluded the use of multiple linear regression to model several traits in combination with each other. This is because collinearity may inflate statistical measurements such as the correlation coefficients and *p*‐values which in turn provides exaggerated effect measures (Belsley et al., 1980; Chen et al., 2024; Mason & Perreault, 1991). Models with multiple testing would also require a statistical correction, which is known to over‐correct for small effect size measures, often limiting the detection of biologically relevant observations (Drezner & Drezner, 2016; Tanner et al., 1997). Secondly, the observed differences between sexes might indicate possible sex‐specific patterns of inheritance which were outside the scope of the present study (Laurentin Táriba, 2023; Wright et al., 2004). Thirdly, although samples were included from the Northern population that likely contains year‐round residents (Uganda), as well as available data for host species (Village weaver) from databases, the sampling number was too low for any meaningful comparison between migratory and resident populations and the comparison to one individual from host species cannot account for the potential existence of greater genetic diversity for clock genes in host species. It should be noted; however, that previous studies have illustrated that neither of the tested genes serve as diagnostic markers to differentiate resident and migratory species (Le Clercq, Bazzi, Cecere, et al., 2023; Lugo Ramos et al., 2017). Lastly, detailed information about the exact timing, direction and distance of migration from, for example, a geolocator (Bazzi et al., 2015; Saino et al., 2015) or isotope study (Bazzi, Galimberti, et al., 2016; Ramudzuli, 2021), was not available and as such a more direct measure for these attributes was not available.

Future studies are needed to expand upon the results of this study. Recommendations include studies on other intra‐African migrants from non‐passerine orders. For the Diederik cuckoo, future work could include a larger sample size for female individuals as well as include individuals from populations that are more likely to show divergence. This could extend to resident birds on islands off the West and East coast of Africa, such as São Tomé and Príncipe or Zanzibar, and migrants that migrate to Oman in the Middle East to breed. More data are also needed on the clock genes of birds that serve as hosts for cuckoos, such as Village weavers, Cape wagtails and Red bishops, in order to fully explore their co‐evolution.

## AUTHOR CONTRIBUTIONS

**L. S. Le Clercq:** Conceptualization (lead); data curation (lead); formal analysis (lead); methodology (lead); validation (lead); visualization (lead); writing – original draft (lead). **V. Phetla:** Data curation (supporting); formal analysis (supporting); writing – review and editing (supporting). **S. T. Osinubi:** Funding acquisition (supporting); investigation (supporting); resources (supporting); writing – review and editing (supporting). **A. Kotzé:** Project administration (supporting); writing – review and editing (supporting). **J. P. Grobler:** Project administration (supporting); supervision (supporting); writing – review and editing (supporting). **D. L. Dalton:** Conceptualization (supporting); funding acquisition (lead); project administration (equal); resources (lead); supervision (lead); writing – review and editing (lead).

## CONFLICT OF INTEREST STATEMENT

The authors have no competing interests to declare.

## Supporting information


Appendix S1.

## Data Availability

Sample details were deposited in the National Centre for Biotechnology Information (NCBI) BioSample database while sequences generated in this study were deposited to the NBCI Nucleotide collection. Accession numbers with associated links to NCBI BioProject PRJNA737185 are listed in (Table S1) (Genbank accession numbers *Clock*: OR909322–51, *Adcyap1*: PP112918–46). Additional data including individual allele data are available from the Zenodo depository at the following link: https://doi.org/10.5281/zenodo.10211358. Allele data have also been added to the Avian Clocks Data database (Le Clercq, 2023b; Le Clercq, Bazzi, et al., 2023e). Methods are available from Protocols.io (Le Clercq et al., 2023c, Le Clercq et al., 2023d).
