# Supplementary material for: Phenotypic correlates between clock genes and phenology among populations of Diederik cuckoo, Chrysococcyx caprius
Source: Ecol Evol. 2024 Jul 31;14(8):e70117. doi: 10.1002/ece3.70117 (PMC11291300; doi:10.1002/ece3.70117)
Supplement: Supplementary file 1 — Appendix S1. [file ECE3-14-e70117-s001.docx]

**SUPPLEMENTARY MATERIAL**

**Table S1:** Details of samples used in the present study along with BioSample and GenBank accession numbers.

| **Catalogue nr** | **BioSample nr** | **Sex** | **Location** | ***Clock*** | ***Adcyap1*** |
| --- | --- | --- | --- | --- | --- |
| 60759CC | SAMN31832894 | male | Uganda | OR909323 | PP112922 |
| 60760CC | SAMN31832895 | male | Uganda | OR909324 | PP112942 |
| 60763CC | SAMN31832896 | male | Uganda | OR909322 | PP112934 |
| 46997CC | SAMN31832897 | male | South Africa | OR909340 | PP112940 |
| 46998CC | SAMN31832898 | male | South Africa | OR909346 | PP112927 |
| 46999CC | SAMN31832899 | male | South Africa | OR909338 | PP112941 |
| 47001CC | SAMN31832900 | male | South Africa | OR909336 | PP112933 |
| 47002CC | SAMN20928759 | female | South Africa | OR909337 | NA |
| 47011CC | SAMN31832901 | male | South Africa | OR909325 | PP112918 |
| 47012CC | SAMN31832902 | male | South Africa | OR909347 | PP112929 |
| 63270CC | SAMN31832903 | male | South Africa | OR909342 | PP112943 |
| 63235CC | SAMN31832904 | male | South Africa | OR909331 | PP112935 |
| 63269CC | SAMN31832905 | male | South Africa | OR909341 | PP112926 |
| 63264CC | SAMN31832906 | female | South Africa | OR909339 | PP112945 |
| 63226CC | SAMN31832907 | female | South Africa | OR909330 | PP112931 |
| 63259CC | SAMN31832908 | female | South Africa | OR909332 | PP112932 |
| 63260CC | SAMN31832909 | female | South Africa | OR909335 | PP112939 |
| 65332CC | SAMN31832910 | female | South Africa | OR909343 | PP112938 |
| 65313CC | SAMN31832911 | female | South Africa | OR909345 | PP112946 |
| 65334CC | SAMN31832912 | male | South Africa | OR909344 | PP112937 |
| 53703CC | SAMN31832913 | male | Nigeria | OR909333 | PP112924 |
| 53713CC | SAMN31832914 | male | Nigeria | OR909350 | PP112925 |
| 53702CC | SAMN31832915 | male | Nigeria | OR909351 | PP112923 |
| 53706CC | SAMN31832916 | male | Nigeria | OR909348 | PP112921 |
| 53705CC | SAMN31832917 | male | Nigeria | OR909334 | PP112944 |
| 53711CC | SAMN31832918 | male | Ghana | OR909349 | PP112920 |
| 60771CC | SAMN31832919 | female | Nigeria | OR909327 | PP112936 |
| 60781CC | SAMN31832920 | female | Nigeria | OR909329 | PP112919 |
| 60776CC | SAMN31832921 | male | Nigeria | OR909328 | PP112928 |
| 60766CC | SAMN31832922 | male | Ghana | OR909326 | PP112930 |

**Table S2**: Details of sequence data retrieved for host species.

| **Host** | **Latin name** | **SRA accession** | ***Clock*** | ***Adcyap1*** |
| --- | --- | --- | --- | --- |
| Village weaver | *Ploceus cucullatus* | SRR17013387 | Q_8_/Q_8_ | 139/141 |
| Cape wagtail | *Motacilla capensis* | SRR5107518 | – | – |
|  |  | SRR5107510 | – | – |
|  |  | SRR5107439 | – | – |
|  |  | SRR5107432 | – | – |
|  |  | SRR5107354 | – | – |
| Southern red bishop | *Euplectes orix* | SRX14011259 | – | – |
|  |  | SRX14011258 | – | – |
|  |  | SRX14010987 | – | – |
|  |  | SRX14010986 | – | – |
|  |  | SRX14010985 | – | – |

**
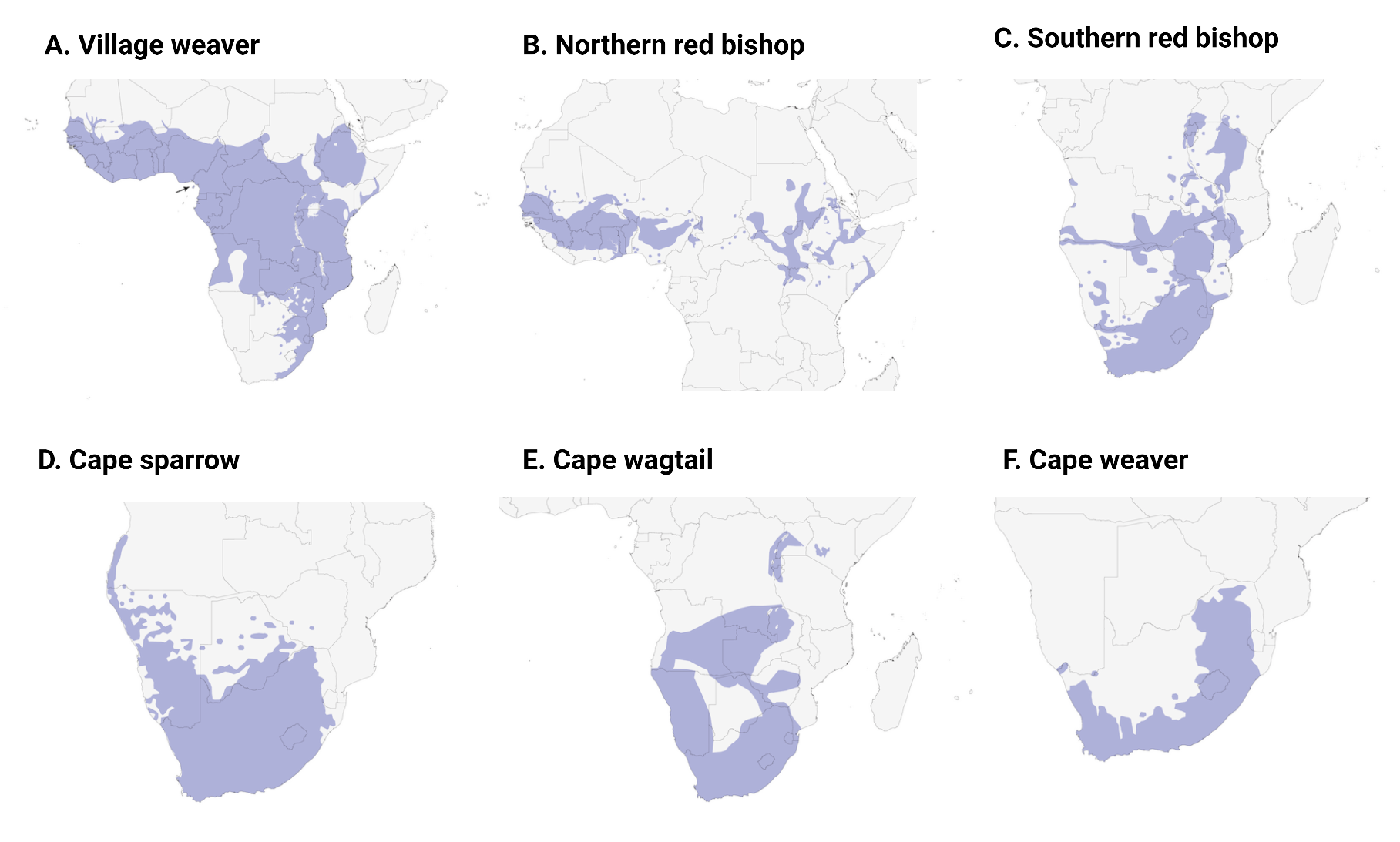
**

**Figure S1**: Host ranges for six bird species which are most commonly parasitised by Diederik cuckoo. In their Northern range they are known to parasitise the Village weaver (**A**) as well as the Northern red bishop (**B**), while in their Southern range they parasitise at least four species. In South-West Africa, they most commonly parasitise the Cape sparrow (**D**) and Cape wagtail (**E**), while in South-East Africa they additionally parasitise the Southern red bishop (**C**) and Cape weaver (**F**).
